# Adaptation to seasonality and the winter freeze

**DOI:** 10.3389/fpls.2013.00167

**Published:** 2013-06-03

**Authors:** Jill C. Preston, Simen R. Sandve

**Affiliations:** ^1^Department of Plant Biology, University of VermontBurlington, VT, USA; ^2^Norwegian University of Life SciencesÅs, Norway

**Keywords:** cold acclimation, freezing tolerance, endodormancy, plant adaptation, seasonality, vernalization responsiveness

## Abstract

Flowering plants initially diversified during the Mesozoic era at least 140 million years ago in regions of the world where temperate seasonal environments were not encountered. Since then several cooling events resulted in the contraction of warm and wet environments and the establishment of novel temperate zones in both hemispheres. In response, less than half of modern angiosperm families have members that evolved specific adaptations to cold seasonal climates, including cold acclimation, freezing tolerance, endodormancy, and vernalization responsiveness. Despite compelling evidence for multiple independent origins, the level of genetic constraint on the evolution of adaptations to seasonal cold is not well understood. However, the recent increase in molecular genetic studies examining the response of model and crop species to seasonal cold offers new insight into the evolutionary lability of these traits. This insight has major implications for our understanding of complex trait evolution, and the potential role of local adaptation in response to past and future climate change. In this review, we discuss the biochemical, morphological, and developmental basis of adaptations to seasonal cold, and synthesize recent literature on the genetic basis of these traits in a phylogenomic context. We find evidence for multiple genetic links between distinct physiological responses to cold, possibly reinforcing the coordinated expression of these traits. Furthermore, repeated recruitment of the same or similar ancestral pathways suggests that land plants might be somewhat pre-adapted to dealing with temperature stress, perhaps making inducible cold traits relatively easy to evolve.

## INTRODUCTION

Since the late Eocene and Oligocene around 47.5 to 26 million years ago (mya) the Earth has experienced dramatic cooling events, resulting in an overall contraction of the tropics, and establishment of novel temperate zones in both northern and southern hemispheres (Zachos et al., 2001; Stickley et al., 2009). In response to this cooling, several ancestrally tropical lineages have successfully diversified outside their ecological zone of origin, becoming adapted to cooler and often more seasonal environments (Latham and Ricklefs, 1993; Sandve and Fjellheim, 2010). However, the fact that less than half the families of modern angiosperms are represented in the temperate zones suggests that adaptations to cold seasonal climates might be difficult to evolve (Ricklefs and Renner, 1994; Donoghue, 2008).

Support for the hypothesis that adaptations to low or freezing seasonal temperatures are relatively hard and/or slow to evolve comes from the fact that climate cooling during the Eocene–Oligocene boundary was associated with large-scale extinctions of both animals and plants (Ivany et al., 2000), and by the apparent complexity of physiological and morphological adaptations to cold (see later sections). However, as an alternative hypothesis, it has been postulated that, since climate cooling has been an ongoing process throughout the Cenozoic, the relatively recent expansion of cold temperate zones has meant that only a minority of plant families have been historically party to selection by cold winters (Fine and Ree, 2006). Thus, there is still much debate about whether different adaptations to extended periods of cold can evolve quickly enough to allow range expansions and/or local adaptation under gradual or rapid climate change conditions (Franks et al., 2007; Cook et al., 2012). The focus of this review is to highlight the major ways in which plants have adapted physiologically to cold seasonal environments, and to synthesize some of the current available data on the genetic basis of these adaptations, with the general goal of understanding the evolutionary lability of cold-season traits.

## PHYSIOLOGICAL AND MORPHOLOGICAL ADAPTATIONS TO SEASONAL COLD

Plant species in both the cold temperate zones and the tropical highlands can experience periods of cold or freezing temperatures that are potentially detrimental to growth and development. However, a key difference between temperate and tropical highland species is the timing and duration of cold, either occurring on a diurnal cycle (tropical highland species) or an annual cycle (temperate species). Despite evidence for overlap in the genetic response to cold (Dhillion et al., 2010; Greenup et al., 2011), the extent to which cold occurs diurnally or seasonally has major implications for the life history strategies that are adopted by plant populations in a given environment (e.g., Teutonico and Osborn, 1995). The focus of this review is physiological adaptation to seasonal cold, i.e., adaptation to the coldest season of the year in temperate climates.

Unlike animals, individual plants are immobile. Thus, in order to reduce the negative effects of winter cold, many temperate plants must synchronize their sensitive reproductive output with favorable environmental conditions of the spring and summer (Bradshaw, 1972; King and Heide, 2009). In the case of spring annuals, germination, reproduction, and senescence occur during the warm seasons. In order to avoid winter growth, spring annual seeds remain dormant during the winter, only to germinate in response to inductive temperatures in the spring (Hemming and Trevaskis, 2011). By contrast, winter annuals set seed and germinate in the fall, overwinter in a vegetative growth state, and flower in the spring. The ability of plants to respond to an extended period of cold to rapidly attain flowering competency is termed vernalization responsiveness (Chouard, 1960). Furthermore, since the aerial vegetative structures (i.e., stems and leaves) of winter annuals are subject to cold, vernalization responsive plants are often, but not always (Rapacz and Markowski, 1999), induced into a state of cold and/or freezing tolerance.

Similar to annuals, temperate perennials vary in their life history strategies for dealing with seasonal cold. In the case of herbaceous perennials, germination, reproduction, and senescence occur during the warm seasons. However, rather than relying only on seed to produce the next generation, herbaceous perennials are capable of secondary rounds of vegetative growth from dormant underground meristems (e.g., rhizomes), which occurs at the conclusion of winter. By contrast, woody perennials such as trees, often delay their flowering for several years until a critical biomass is achieved (Rohde and Bhalerao, 2007). As in the case of winter annuals, temperate herbaceous perennials are often responsive to vernalization, and can tolerate chilling and frost. Furthermore, in addition to cold tolerance, many temperate and boreal trees are able to protect new growth from harsh winter conditions by becoming dormant prior to winter (endodormancy; Lang et al., 1987; Howe et al., 2003; Campoy and Egea, 2011). Recent studies on the genetic basis of these varied adaptations to winter cold offer exciting opportunities to understand constraints on plant transitions from the tropical to the temperate zone, and *vice versa*. This is particularly relevant in the face of current and projected changes to our climate. The following sections will focus on the evolution and genetic basis of three important physiological cold adaptations: cold acclimation (i.e., the seasonal acquisition of cold and freezing tolerance), endodormancy, and vernalization responsiveness. However, first we will consider what is currently known about the phylogenetic pattern of these traits across seed plants, and their relationships to climate.

## PHENOTYPIC CORRELATIONS AND THE PHYLOGENETIC DISTRIBUTION OF COLD ADAPTIVE TRAITS

In temperate plants that experience prolonged cold to sub-zero winter temperatures, above ground tissues are susceptible to delayed growth and damage by frost. Thus, high latitude plants that undergo endodormancy (woody perennials), or are responsive to vernalization (herbaceous annuals and perennials), are often also able to induce cold tolerance through a process known as cold acclimation (Howe et al., 2003; see Cold Acclimation and Cold Tolerance). For example, non-vernalization responsive (spring) wheat varieties generally have lower freezing tolerance than vernalization responsive (winter) wheat, and in winter wheat length of vernalization requirement is positively correlated with speed of cold acclimation (Prasil et al., 2004). Furthermore, in temperate trees such as *Pinus contorta* and Douglas-fir (*Pseudotsuga menziesii*), elevation and distance from warmer ocean climates are strongly associated with the timing of growth cessation and endodormancy and the temperature required to induce cold acclimation and subsequent freezing tolerance (Campbell and Sugano, 1979; Howe et al., 2003). A similar trend has been found between length of vernalization needed to elicit flowering and continental-oceanic gradients in *A. thaliana* (Lewandowska-Sabat et al., 2012).

Despite the correlation between cold adaptive traits, there are examples of endodormant and vernalization responsive plants that cannot induce cold tolerance, and *vice versa*. For example, *Thuja plicata* and *Tsuga heterophylla* both acclimate to cold, but do not experience endodormancy (Silim and Lavender, 1994), several trees undergo endodormancy without acclimating to cold (Kramer and Kozlowski, 1979), and several vernalization-responsiveness cereal cultivars are considered cold-sensitive (Tester and Bacic, 2005). The lack of a strict association between endodormancy/vernalization responsiveness and cold acclimation might be explained by the fact that low non-freezing temperatures can be detrimental to young bud development without affecting growth of other plant structures. For example, in plants adapted to, or derived from, subtropical climates, synchronization of bud development with warm conditions might be enough to escape the negative effects of occasional low winter temperatures, without the additional need for cold/freezing tolerance. Alternatively, boreal plants that experience sub-zero temperatures for large parts of the year might benefit from constitutive freezing tolerance, negating the importance of cold acclimation.

**FIGURE 1:**
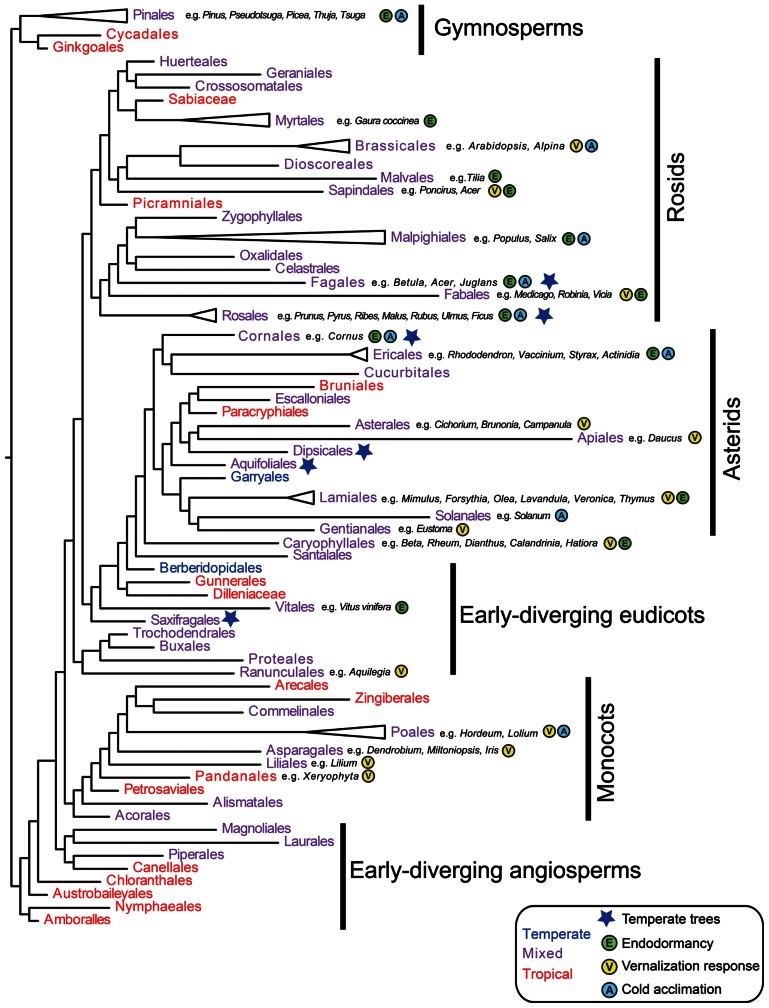
**Evolution of cold adaptive traits in seed plants.** Relationships among major seed plant orders are inferred using representative taxa from Smith et al. (2011) in phylomatic (Webb and Donoghue, 2005). Orders are color-coded as primarily temperate (blue), broadly distributed, or primarily tropical (red) based on the APG website (Stevens, 2001 onward). Blue stars indicate orders where trees are primarily temperate based on Ricklefs (2005). Evidence for endodormancy (E), cold acclimation/freezing tolerance (A), and vernalization responsiveness (V) are denoted for each order with example species. Since most species have not been tested for cold adaptations, absence of data does not necessarily indicate absence of traits. However, since cold climates arose after major radiations in seed plants, presence data (based on Krug, 1991; De la Rosa et al., 2000; Kawamata et al., 2002; Wilson et al., 2002; Karlson et al., 2004; Streck and Schuh, 2005; Lopez and Runkle, 2006; Fausey and Cameron, 2007; Kalberer et al., 2007; Mewes and Pank, 2007; Rohwer and Heins, 2007; Svendsen et al., 2007; Padhye and Cameron, 2008, 2009; Pietsch et al., 2009; Zlesak and Anderson, 2009; Biasi et al., 2010; Byard et al., 2010; Ghelardini et al., 2010; Kaymak and Guvenc, 2010; Kubota et al., 2010; Lenahan et al., 2010; Rantasen and Palonen, 2010; Caffarra et al., 2011; Cave et al., 2011; Charrier et al., 2011; Dogramaci et al., 2011; Lin et al., 2011; Adhikari et al., 2012; Andreini et al., 2012; Bilavcik et al., 2012; Diaz-Riquelme et al., 2012; Nishitani et al., 2012; Sanchez-Perez et al., 2012; Whitman and Runkle, 2012; Alessandro et al., 2013; Guzy-Wrobelska et al., 2013; Jones et al., 2013; Mojtahedi et al., 2013) indicates multiple origins of cold adaptive traits across the phylogeny.

As outlined in the introduction, angiosperm families containing temperate species are less common than families confined to the tropics (Ricklefs and Renner, 1994; Donoghue, 2008). However, temperate taxa are distributed throughout the seed plant phylogeny (Ricklefs, 2005; **Figure 1**). Thus, the timing of angiosperm diversification relative to global Eocene cooling events suggests numerous independent origins of temperate seed plants (Wang et al., 2009b). How many of these major lineages evolved physiological adaptations to seasonal cold? With the exception of early-diverging angiosperms and several tropical gymnosperms for which there are no or limited experimental data, a broad literature search suggests that cold acclimation, endodormancy, and vernalization responsiveness have evolved in all major seed plant clades (**Figure 1**). Thus, at a broad phylogenetic scale, adaptations to cold might be relatively easy to evolve. In the following sections, we review available genetic data to determine whether multiple independent origins of cold traits can be explained by the modification of pre-adapted pathways (e.g., exaptations, *sensu* Gould and Vrba, 1982), and/or represent novel evolutionary innovations. We also suggest future studies that could be carried out to determine the potential for evolution of seasonal cold traits on shorter timescales and in response to current global change.

## COLD ACCLIMATION AND COLD TOLERANCE

**FIGURE 2:**
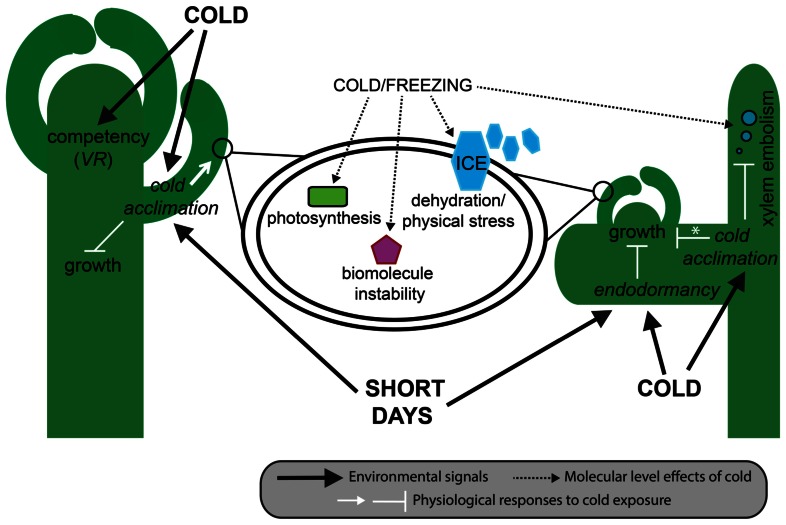
**Effect of cold on plant phenotype.** In herbaceous plants (left) such as *A. thaliana* and wheat, cool autumnal temperatures induce cold acclimation mostly in young leaves, and the acquisition of floral competency in the shoot apical meristem. In *A. thaliana*, cold acclimation is also induced by short days, and results in a decrease in growth rate through the gibberellic acid pathway, and an increase in freezing tolerance within cells (middle). In woody plants (right) such as pines and birch, endodormancy can be induced as early as late summer and results in the complete cessation of meristematic mitosis. As in herbaceous plants, cool temperatures lead to cold acclimation and a gradual increase in freezing tolerance, sometimes (e.g., deciduous trees) but not always (e.g., evergreen trees) resulting in a reduction in whole-plant growth. At the cellular level, freezing tolerance results from the ability of cells to deal with dehydration, ice crystal formation, biomolecule instability, and disruption to photosynthesis. At the whole-plant level, some freezing tolerant woody plants are able to deal with the increased likelihood of embolism resulting from bubbles formed when dissolved gases are released from frozen xylem sap. Asterisk denotes species-level differences in growth responses to cold acclimation. VR, vernalization responsiveness.

Cold tolerance is a highly complex trait that encompasses both the ability to tolerate the direct effects of low temperatures on plant function and the indirect effects of ice formation in and surrounding the plant. Direct effects of cold conditions change biological thermodynamic processes, biomolecule stability and function, and alter normal cellular processes such as photosynthesis, inter- and intracellular transport, and the balance between production and neutralization of toxic reactive oxygen species (ROS; Thomashow, 1998; **Figure 2**). By contrast, indirect effects of ice formation that occur during sub-zero temperatures create a different kind of cellular stress. First, extracellular ice formation depletes available water in and around cells, affecting normal water dependent processes and inducing freezing dehydration with associated cell membrane disruption (Steponkus et al., 1998). Second, large ice crystals grow at the expense of the formation of small new crystals, a process referred to as ice recrystallization (Capicciotti et al., 2012). This process generates large and potentially damaging expanding crystals in extracellular spaces. Although most species have some innate tolerance to a sudden exposure to cold, many temperate species have evolved the ability to gradually increase their freezing tolerance during extended periods of cold, but non-freezing, temperatures and changing photoperiod during autumn (Thomashow, 1999; Catalá et al., 2011). This inducible process is referred to as cold acclimation, and ultimately leads to healthy plants that can successfully reproduce the following spring (**Figure 2**).

### MOLECULAR AND PHYSIOLOGICAL CHANGES ASSOCIATED WITH COLD ACCLIMATION AND COLD TOLERANCE

Cold acclimation involves major changes in the biochemical and physiological state of the plant, improving low temperature stress tolerance. In general, proteins and compounds with various protective functions are increased, while photosynthesis and several other metabolism-related biochemical pathways are suppressed (e.g., Fowler and Thomashow, 2002; Lee et al., 2005; Rudi et al., 2010; Winfield et al., 2010; **Table 1**). Although cold acclimation genes and genetic pathways can vary widely between species, some molecular and physiological changes seem to be similar across major angiosperm clades (reviewed in Sandve et al., 2011).

**Table 1:** Genes and pathways regulated during cold acclimation.

**Class**	**Subclass**	**Regulation**	**Function**	**Reference**
Protective	Antioxidant	Up	Free oxygen radical regulation	Winfield et al. (2010)
	Chaperones	Up	Biomolecule protection/stabilization	Carvallo et al. (2011)
	Dehydrins/LEA	Up	Unknown	Hanin et al. (2011)
	Proline	Up	Osmoregulation	Janská et al. (2011)
	GABA	Up	Osmoregulation	Janská et al. (2011)
	Ice interacting, e.g., *LpIRI-a/b*	Up	Reduce freezing point, inhibit ice recrystallization	Sidebottom et al. (2000); Griffith and Yaish (2004), Winfield et al. (2010); Zhang et al. (2010), Janská et al. (2011)
Protective/signaling	Carbohydrate metabolism/starch degradation	Up	Stabilize membranes, osmoregulation, signaling	Maruyama et al. (2009); Janská et al. (2011)
Lipid membrane	Lipid membrane remodeling, e.g., *AtSFR2*	Up/down	Non-bilayer formation, membrane stabilization	Moellering et al. (2010)
Metabolism	Homeostatic	Down	Metabolic and energetic control	Huner et al. (1993); Fowler and Thomashow (2002), Li et al. (2004)

The ability to manipulate ice formation has arisen multiple times throughout angiosperm evolution, and is achieved either by decreasing the freezing point (thermal hysteresis) or by inhibiting ice recrystallization (Griffith and Yaish, 2004; Byard et al., 2010; **Figure 2**). Although thermal hysteresis is commonly found in plants, it is not affected much by cold acclimation (Urrutia et al., 1992); ice recrystallization inhibition is believed to be more important for plant cold acclimation (Griffith and Yaish, 2004; **Table 1**). Ice recrystallization inhibition is found in species of many plant lineages (Doucet et al., 2000) and is caused by a range of diverse proteins, including beta-1,3-glucanases, WRKY proteins, chitinases, and thaumatin-like proteins (Griffith and Yaish, 2004 and references therein). For example, in eudicots the carrot (*Daucus carota*) polygalacturonase inhibitor protein inhibits ice recrystallization and decreases the freezing point; its expression in tobacco (*Solanum tabacum*) and *A. thaliana* results in inhibition of ice recrystallization and increased freezing tolerance (Worrall et al., 1998; Meyer et al., 1999). In monocots, a different Pooideae (Poaceae)-specific inhibitor of ice recrystallization-protein (IRIP) family shows strong inhibition of ice recrystallization (Sidebottom et al., 2000), which has been shown to increase freezing tolerance *in planta* (Zhang et al., 2010).

Freezing induced cell dehydration is another factor plants have to deal with during winter (Uemura et al., 1995; **Figure 2**). During cold acclimation plants gain the ability to modify membrane stability, which involves both changes to the cell membrane lipid content (Li et al., 2004; Moellering et al., 2010) and production of membrane-interacting protective compounds, such as proline and a diversity of carbohydrates (Fujikawa et al., 1999; Hisano et al., 2004; Valluru and Van den Ende, 2008; **Table 1**). Manipulation of lipid metabolism and membrane lipid composition in transgenic plants has improved freezing and chilling tolerance in tobacco (Khodakovskaya et al., 2006), poplar (*Populus* sp.; Zhou et al., 2009), and tomato (*S. lycopersicum*; Domínguez et al., 2010).

In addition to proteins and compounds with direct protective action in cold, modulation of photosynthetic processes is a common cold acclimation response among angiosperms (**Figure 2**). During photosynthesis, light energy is absorbed and converted to chemical energy in thylakoid membranes of chloroplasts, and then used for CO_2_-fixation in the Calvin cycle. Absorption of light by photosystem II (PSII) normally leads to light-induced damage of the PSII, a process referred to as photoinhibition, which is counteracted by a PSII damage repair mechanism that restores PSII function (Hetherington et al., 1989; Aro et al., 1993, 2005; Melis, 1999; Bascuñán-Godoy et al., 2012). However, if the level of absorbed light energy greatly exceeds that of the consumed chemical energy this will impair PSII damage repair and accelerate photoinhibition (Takahashi and Murata, 2008), with potential detrimental consequences for plant growth (Melis, 1999). Low temperatures promote increased photoinhibition (Hetherington et al., 1989), and to minimize photoinhibition-associated damage, higher plants undergo photosynthetic acclimation during cold acclimation either by increasing the energy demand through increased carbon assimilation and carbon metabolism (Huner et al., 1993), dissipation of excess excitation energy as heat (Dall’Osto et al., 2005), or improving the PSII repair machinery (Bascuñán-Godoy et al., 2012; **Table 1**). Photoinhibition is also detrimental to the entire cell due to the associated increase of ROS (Krause, 1988). Photoinhibition has been shown to regulate the expression of genes in cold acclimation (Gray et al., 1997), hence, a plants’ freezing tolerance is inherently linked to temperature-induced photoinhibition. Variation in the capacity for photosynthetic acclimation during cold acclimation is correlated with genotypic differences in winter survival and freezing tolerance of grasses (Rapacz et al., 2004). Moreover, the C-REPEAT binding factor (CBF) pathway has been shown to alleviate photoinhibition in autumn conditions (Yang et al., 2010).

In woody species, freezing temperatures can disrupt whole-plant functioning by limiting long-distance water transport in the xylem (Sperry et al., 1994; Améglio et al., 2001). This is particularly true following xylem embolisms (**Figure 2**), which are often induced by freeze–thaw cycles in regions that experience freezing winter nights and above freezing winter days, such as North Africa, the Mediterranean region of Europe, and southern parts of North America (Cavendar-Bares et al., 2005; Meideros and Pockman, 2011). Although variation in resistance to embolism can be explained by differences in vessel diameter and architecture (Sperry et al., 1994; Améglio et al., 2001), cold acclimation has been found to reduce xylem embolisms in oak (*Quercus*) and several conifers (Hammel, 1967; Sperry et al., 1994; Cavendar-Bares et al., 2005). Furthermore, it is hypothesized that some woody plants can repair winter embolism; the mechanisms underlying repair and induced resistance are largely unknown (Cavendar-Bares et al., 2005).

### SENSING AND SIGNALING COLD

How low temperature is sensed and then signaled to the cell nucleus is generally not well understood. The best-studied temperature sensing mechanism is membrane fluidity. Membranes surrounding cells, mitochondria, and chloroplasts consist of a lipid bilayer, and low temperatures causes lipid membranes to become more rigid (Alonso et al., 1997). Chemically induced membrane rigidity results in cytoskeleton changes, increased Ca^2^^+^ influx to the cell, and changes in activity of certain protein kinases, which ultimately result in transcription of cold-induced genes that artificially mimic the process of cold acclimation and improve freezing tolerance of plants (Örvar et al., 2000; Sangwan et al., 2001). In addition to cell membrane changes, abscisic acid (ABA; Llorente et al., 2000; Xiong et al., 2001) and ROS (Lee et al., 2002) accumulation in warm conditions has been shown to initiate processes similar to cold acclimation, resulting in increased freezing tolerance.

Recently, a molecular model of cold signaling, linking cold sensing and transcription, has been put forward (Doherty et al., 2009). The model includes four components; Ca^2^^+^, calcium modulated proteins (calmodulins), calmodulin binding transcriptional activators (CAMTAs), and cold responsive transcription factors. Low temperatures increase the Ca^2^^+^ influx (probably as an indirect response to more rigid membranes) and activate calmodulin proteins, which subsequently activate other Ca^2^^+^-unresponsive CAMTA proteins essential for cold acclimation. The *A. thaliana* genome contains six CAMTA genes with calmodulin binding- and DNA binding CG-1 (CGCG) domains (Bouché et al., 2002). Absence of the CG-1 element in promoters of cold responsive transcription factors (both CBF and non-CBF), leads to a decrease in their transcript levels of up to 40–50% (Doherty et al., 2009). Loss-of-function CAMTA gene mutants are unable to acclimate to cold and drought, and so are sensitive to freezing (Doherty et al., 2009; Pandey et al., 2013). It should be noted that the validity of this model with respect to the role of calmodulin as Ca^2^^+^ signal sensors has not been experimentally tested.

### TRANSCRIPTIONAL REGULATION OF THE COLD ACCLIMATION PROCESS

At least 50–60 transcription factors are known to be important in initiation of cold acclimation in *A. thaliana*, including members of the AP2, MYB, MYC, bZIP, and Zn-FINGER transcription factor families, but relatively little is known about their downstream targets (Fowler and Thomashow, 2002; Lee et al., 2005; Vogel et al., 2005; Knight et al., 2009). The only well characterized cold acclimation pathway in plants is the INDUCER OF CBF EXPRESSION (ICE)-CBF-COLD-RESPONSIVE (COR) cold response pathway (Gilmour et al., 1988; Chinnusamy et al., 2003; Stockinger et al., 2007). During plant chilling, expression of *ICE* genes triggers rapid (~15 min) transient up-regulation of CBFs (Chinnusamy et al., 2003; Dong et al., 2006), which together directly or indirectly regulate approximately 30% of all cold-induced transcriptional changes (Vogel et al., 2005; Svensson et al., 2006; Van Buskirk and Thomashow, 2006; **Table 1**). The initial ICE-CBF regulatory switch was described in *A. thaliana*, but data suggest it is functionally conserved in apple (*Malus domestica*, Rosaceae), and (at least partially) in grasses, subfamily Pooideae (Chinnusamy et al., 2003; Badawi et al., 2008; Feng et al., 2012). Cold-induced CBFs are found in species across all land plant lineages (e.g., Skinner et al., 2005; Xiong and Fei, 2006; Pennycooke et al., 2008), and their protein products bind to CCGAC core motifs in the promoters of diverse cold and dehydration responsive genes, including themselves (Liu et al., 1998; Novillo et al., 2004; Stockinger et al., 2007).

In addition to cold, *CBF* transcription is affected by photoperiod. Expression of *A. thaliana CBF*s fluctuates on a diurnal basis, peaking around 8 h after the dawn zeitgeber (Fowler et al., 2005). Under long day conditions, *CBF* gene expression is repressed by the action of PHYTOCHROME B (PHYB), PHYTOCHROME-INTERACTING FACTOR 4 (PIF4) and PIF7 (Lee and Thomashow, 2012). By contrast, under short days, reduced mRNA levels and stability of *PHYB*, *PIF4*, *PIF7* and their protein products, respectively, result in the derepression of *CBF* transcription (Lee and Thomashow, 2012). Although the circadian regulation of *CBF*s is not well understood, the differential regulation of *CBF*s under short versus long days provides a secondary mechanism by which freezing tolerance can be timed to coincide with winter.

The plant hormone ABA is a major player in regulating genes involved in plant stress response through the transcriptional activation of ABA-dependent transcription factors (Shinozaki and Yamaguchi-Shinozaki, 2000). The importance of ABA in freezing tolerance is debated (Gusta et al., 2005), but much evidence supports a role for ABA in cold acclimation under natural conditions. First, endogenous ABA-levels increase in *A. thaliana* and wheat (*Triticum aestivum*) during low temperature exposure (Cuevas et al., 2008; Shakirova et al., 2009). Second, application of exogenous ABA enhances freezing tolerance in whole plants (Chen and Gusta, 1983; Mantyla et al., 1995) and calli (Dallaire et al., 1994). Third, many genes expressed during cold acclimation are regulated by ABA, including the CBF genes (Hoth et al., 2002; Knight et al., 2004; Cuevas et al., 2008; Kobayashi et al., 2008; Shakirova et al., 2009; Agarwal and Jha, 2010). Involvement of ABA in cold transcriptional regulation is observed in bryophytes (Bhyan et al., 2012), monocots, and eudicots (reviewed in Gusta et al., 2005).

### CONSERVATION AND DIVERSIFICATION OF COLD ACCLIMATION AND COLD TOLERANCE

Despite independent evolution over hundreds of millions of years, some pathways and mechanisms involved in cold acclimation are similar between species of bryophytes, monocots, and eudicots (see above). This could be interpreted as evidence for conservation of ancestral cold response pathways from the earliest land plant through the diversification of all major land plant lineages. An alternative interpretation is that similarities in cold responses across land plant lineages are due to genetic parallelisms. The latter could occur if an ancient stress response pathway was recruited to cold acclimation and cold/freezing tolerance multiple times independently. Predictions of this hypothesis include substantial overlap between the cold acclimation/tolerance and other stress pathways.

One potential pathway that might have been recruited for cold acclimation and cold/freezing tolerance is the drought tolerance pathway. All land plants are constantly battling to minimize water loss at the atmosphere–plant boundary and prevent cellular dehydration. Adaptations to withstand dehydration were probably some of the main evolutionary innovations when plants moved from aquatic to terrestrial life ~500 mya; hence basic molecular responses to dehydration can be assumed to have a common ancestry in all land plants. Interestingly, many key cold acclimation responses are tightly linked to dehydration. For example, in *A. thaliana* gene expression correlations of 0.15–0.30 are found between response to dehydration stresses (drought, salt, and osmotic stress) and cold stress (Swindell, 2006). This strong molecular connection between dehydration-like stress responses and cold can only be explained by the control of these responses through non-specific stress triggers, perhaps by cellular redox states, or some other shared signaling mechanisms.

Following this logic, distantly related cold or freezing tolerant species are predicted to have adapted to cold through changes in partially overlapping molecular pathways, resulting in the recurring recruitment of (a few) similar pathways. If this hypothesis holds, we would expect relatively similar initial transcriptional responses and more diverse downstream molecular changes between species with independent adaptations to cold. Comparative transcriptome analyses have shown conserved expression profiles during cold acclimation in potato (*S. tuberosum*) and *A. thaliana*, despite over 100 mya of independent evolution (Carvallo et al., 2011). However, more comprehensive comparative studies of stress responses among species are needed to better understand the patterns of transcriptional conservation over macro-evolutionary time scales.

Intriguingly, cold acclimation has also been demonstrated in green algae (Nagao et al., 2008), but the alga process does not respond to ABA as many land plants do, suggesting involvement of different pathways. Algae do contain AP2 domain encoding genes like the CBF transcription factors, but it is not clear if these transcription factors are involved in cold acclimation. More detailed studies in algae will enable us to understand if the basic molecular modules of plant cold acclimation evolved as early as in an aquatic land plant ancestor.

## ENDODORMANCY

**FIGURE 3:**
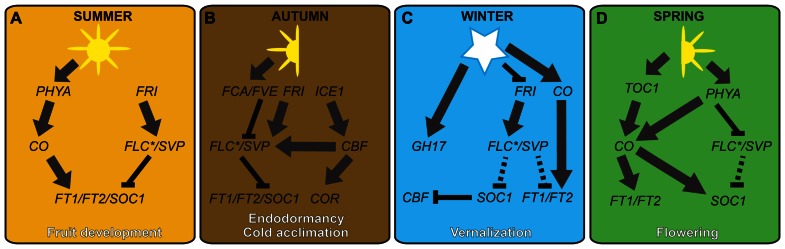
**Hypothetical model showing possible genetic links between cold-regulated growth and reproduction in temperate plants.** Expression levels of *FT*- and *SOC1*-like flowering integrator genes are maintained at moderate levels in summer by the antagonistic action of long day and warm temperature-regulated genes **(A)**. During cooler temperatures of the autumn, the *ICE-CBF-COR* cold acclimation pathway is initiated, resulting in a high level of flowering repressors, such as *FLC*- and *SVP*-like genes. Although *SVP*-like gene transcription is dampened by the negative action of FCA- and FVE-like proteins, levels are high enough to work with *FLC* to repress *FT*-like and *SOC1*-like genes, resulting in endodormancy in woody perennials **(B)**. Freezing winter temperatures negatively regulate *FRI*- and *FLC*-like genes, or functional equivalents, resulting in the derepression of *FT*- and *SOC1*-like genes, and the subsequent negative regulation of cold acclimation genes **(C)**. However, despite up-regulation of *FT*-like genes in leaves, callus plugs in the shoot apices hinder the floral transition. During warm conditions of the spring, the combination of callus plug decay and long day regulation of *FT*-like genes results in bud flush and the induction of flowering **(D)**. Unbroken and broken lines represent strong and weak interactions, respectively.

Meristem dormancy is a common phenomenon in plants and has been linked to variation in a number of developmental genes (reviewed in Doust, 2007; Domagalska and Leyser, 2011). The most common type of meristem dormancy derives from the ability of the main axis to suppress the outgrowth of axillary meristems (apical dominance or paradormancy), and is a major determinant of architecture and growth habit across higher plants (Lang et al., 1987). By contrast, endodormancy is specific to temperate woody perennials, being shaped by both internal factors and seasonal fluctuations in both temperature and photoperiod (Chuine et al., 2001; reviewed in Campoy and Egea, 2011; **Figures 2** and **3**). Although closely linked to cold acclimation, endodormancy is a distinct physiological process that is sensitive to, but does not require, cold or other external factors to be induced (Faust et al., 1995). Endodormancy can be defined as dormancy under conditions that are conducive to growth (Dogramaci et al., 2011). By contrast, bud flush, which is induced after endodormancy is broken and mitotic division reinitiated, relies on a particular regime of cold followed by warm temperatures, the duration and timing of which is cultivar/species specific, and is often correlated with latitude (Saure, 1985; Erez and Couvillon, 1987; Naor et al., 2003; Campoy et al., 2011).

Despite inherent difficulties in working with woody species, research on both temperate gymnosperms and angiosperms, such as apple (*M. domestica*), apricot (*Prunus armeniaca*), poplar (*Populus trichocarpa*), hybrid aspen (*Populus tremula* × *Populus tremuloides*), and woody spurge (*Euphorbia esula*), have revealed many genes and gene networks underlying endodormancy and bud break (Dogramaci et al., 2011; Hsu et al., 2011; Karlberg et al., 2011; reviewed in Campoy and Egea, 2011; **Figure 3**). Available data suggest a complex interplay between the circadian clock and ABA (autonomous), gibberellic acid, photoperiod, and temperature pathways (reviewed in Campoy and Egea, 2011). In species such as poplar and grape (*Vitis vinifera*), photoperiod and temperature appear to be major determinants of the timing of bud set (defined as bud formation and growth cessation) and endodormancy (Howe et al., 2003; Rohde et al., 2011). By contrast, species such as *Pinus contorta* that can become endodormant as early as late summer use alternative cues such as drought and/or node number (Chuine et al., 2001; Howe et al., 2003). The endodormancy model presented below takes into account the former; future work is required to determine the genetic basis for summer onset endodormancy.

### GENETIC MODEL FOR BUD SET AND ENDODORMANCY

Although not all aspects of autumn endodormancy have been worked out, the most plausible genetic model is that bud set and endodormancy are controlled largely through the differential regulation of *FLOWERING LOCUS T* (*FT*) by photoperiod and/or temperature (**Figure 3**). According to research on poplar and *A. thaliana*, long summer days stabilize the CONSTANS (CO) protein through the action of the light-absorbing protein complex GIGANTIA (GI)/FLAVIN KELCH F BOX (FKF1; Sawa et al., 2007; Song et al., 2012). Late in the day, *GI/FKF1* also acts to degrade *CYCLING DOF FACTORs* (*CDFs*), which are transcriptional repressors of *CO*, and CO protein levels are stabilized through the action of another light-absorbing protein PHYTOCHROME A (PHYA; Yanofsky and Kay, 2002; Fornara et al., 2009; Andres and Coupland, 2012). In turn CO up-regulates the major flowering pathway integrator *FT*, resulting in meristem outgrowth and the development of leaf and branch initials (bud set; Samach et al., 2000). It is postulated that FT-regulated summer growth is mediated by the action of *AINTEGUMENTALIKE1* (*AIL*) genes that are the direct targets of FT. Unlike *A. thaliana*, *AIL* genes in poplar and hybrid aspen positively control growth regulators such as D-type cyclins, suggesting that these genes have been recruited to the endodormancy pathway (Karlberg et al., 2011).

In contrast to the summer growth model, the poplar endodormancy model posits that as temperatures become cooler with the onset of autumn, expression of circadian clock genes, such as *TIMING OF CAB EXPRESSION1* (*TOC1*) and *PSEUDO-RESPONSE REGULATOR*s (*PRR*s), is repressed so that *CO* expression is no longer induced (Mas et al., 2003; reviewed in Imaizumi, 2010; **Figure 3**). The ability of temperature to regulate circadian clock gene expression is still being investigated. However, evidence suggests that diurnal hot/cold cycles can replace photoperiod cycles for entrainment of the circadian clock (Salomé and McClung, 2005; Yamashino et al., 2008; Zuther et al., 2012). Furthermore, the reduced day length of autumn means that PHYA is no longer able to repress the expression of dark-regulated genes that reduce CO protein stability, such as *CONSTITUTIVE PHOTOMORPHOGENIC1* (*COP1*) and *SUPPRESSOR OF PHYTOCHROME A 1* (*SPA1*; Jang et al., 2008; Zuo et al., 2011). In addition to CO-dependent reduction of *FT* levels in the autumn, it is speculated that poplar *FT* is further repressed by the action of the *SHORT VEGETATIVE PHASE* (*SVP*)-like genes *DORMANCY ASSOCIATED MADS-BOX 5* (*DAM5*) and *DAM6*; reviewed in Campoy and Egea, 2011). Thus, since poplar *FT* potentially mediates signals from the leaves to shoot apical meristems, *SVP*-like mediated *AIL* gene repression might cause the autumnal cessation of bud growth, which marks the start of leaf fall and the beginning of cold acclimation (Karlberg et al., 2011; **Figure 3**). Further testing of the genetic interactions between these genes and their protein products will be required to validate this model.

### GENETIC MODEL FOR ENDODORMANCY RELEASE AND BUD FLUSH

Endodormancy occurs either in the absence of cold or with intermittent cold, whereas release of endodormancy occurs in response to continuous above-freezing temperatures of the late autumn and winter (Vegis, 1964). As outlined above, levels of the growth activator/floral pathway integrator protein FT are reduced under short days from autumn onward due to low levels of CO (Yoo et al., 2007; Joon Seo et al., 2013). However, during chilling conditions of the late autumn and early winter, repression of the *FT* inhibitor SVP is also reduced, resulting in a potentially moderate increase of *FT* during winter (Lee et al., 2007; **Figure 3**). In addition to *A. thaliana SVP*, repression in response to prolonged cold was recently demonstrated for the *SVP*-like *DAM6* genes in Japanese apricot (*Prunus mume*; Sasaki et al., 2011). Moreover, *SVP*-like genes have been implicated in induction of endodormancy in leafy spurge (*Euphorbia esula*; Horvath et al., 2008). Thus, temperature regulation of *SVP*-like genes could be a common mechanism for endodormancy release during the winter through the negative regulation of *FT*-like genes.

Rinne et al. (2011) recently suggested a potential complicating factor regarding the involvement of *FT* in endodormancy release in temperate and boreal trees. During cool autumnal temperatures, mobile signals are potentially blocked from entering hybrid aspen shoot apices due to the presence of callus plugs (Rinne et al., 2001). However, during winter freezing temperatures, callus plugs are gradually removed through the activation of gibberellic acid responsive genes that regulate GH17 proteins, the latter being associated with lipid bodies that help to breakdown callose (Rinne et al., 2011). Since FT in *A. thaliana* is a mobile protein that travels from leaves to shoot apices, the formation of callus plugs is one mechanism that might delay FT signaling in the shoot apex during autumn. An explicit test of this hypothesis will be required to determine whether FT is mobile in hybrid aspen and other temperate trees. Furthermore, if FT levels do gradually increase in the shoot apex during the winter it will be interesting to determine whether this influences the timing of endodormancy release, bud flush, or both.

### BUD FLUSH

Endodormancy affects both vegetative and reproductive meristems through the negative regulation of *FT*. However, since FT is a positive regulator of inflorescence meristem genes, it has been unclear how vegetative meristems maintain their identity during bud flush. As a possible solution to this puzzle, Hsu et al. (2011) recently demonstrated that poplar contains two *FT* paralogs, *FT1* and *FT2*, that are differentially expressed both spatially and temporally in response to temperature (both genes) and daylength (*FT2*; **Figure 3**). Whereas expression of *FT1* peaks during winter in leaves, shoots, vegetative buds, and reproductive buds, *FT2* expression is highest during spring, and is confined to leaves and reproductive buds (Hsu et al., 2011). The sequential action of *FT1* and *FT2* on axillary meristems in winter and spring, respectively, results in discrete zones of floral growth and vegetative growth along lateral shoots. A similar partitioning of *FT*-like gene function has been described for the vernalization response of sugar beet (*Beta vulgaris*; Pin et al., 2010; see later section).

### EVOLUTION OF ENDODORMANCY

Like tree habit, endodormancy has multiple independent evolutionary origins, being found in diverse lineages of both gymnosperms and angiosperms (**Figure 1**). Recent studies in poplar suggest that duplication and diversification of *FT*-like genes has been important for the periodic growth of vegetative and inflorescence structures along the shoot axis (Hsu et al., 2011). However, it is unclear whether diversification of *FT*-like genes contributed to the evolution of endodormancy *per se*. Intriguingly, functional analyses of the closest *FT* homologs in spruces (*Picea* sp.) and pines (*Pinus* sp.) suggest that the positive role of *FT* in flowering time evolved after the split of gymnosperms and angiosperms (Klintenäs et al., 2012). Constitutive expression of spruce *FT*-like genes in *A. thaliana* results in late flowering phenotypes, suggesting that the gymnosperm *FT*-like genes repress flowering similar to the *A. thaliana FT* paralog *TFL1* (Klintenäs et al., 2012). In Norway spruce (*Picea abies*), *PaFTL1* and *PaFTL2* are up-regulated under short days and spring conditions, respectively (Gyllenstrand et al., 2007; Asante et al., 2011; Karlgren et al., 2011). Together these studies suggest independent recruitment of *FT*-like genes in angiosperm and gymnosperm bud set and bud burst. Similar studies in other plants will be required to determine the prevalence of *FT*-like gene involvement in the evolution of endodormancy. Furthermore, future research is needed to determine the nature of regulatory evolution in the *FT* gene family.

## VERNALIZATION RESPONSIVENESS

Vernalization is the process by which an extended period of cold makes plants competent to flower (Chouard, 1960). In other words, vernalization responsive individuals will flower earlier under inductive conditions (long days and warm temperatures) when those conditions are preceded by a prolonged exposure to cold. This allows plants to synchronize flowering with favorable conditions of the spring (Amasino, 2010). Unlike endodormancy, shoot apices of vernalized plants continue to undergo some level of mitotic division, so that vegetative growth is maintained. In addition, vernalization is distinct from seed stratification, the latter being the release of seed dormancy through chilling (reviewed in Finch-Savage and Leubner-Metzger, 2006).

Extensive variation for vernalization responsiveness is found within many lineages of angiosperms, and is associated with both latitudinal clines and temperature/precipitation variables (Briggs and Walters, 1997; Stinchcombe et al., 2004; Franks et al., 2007; Samis et al., 2008; Kim et al., 2009; Méndez-Vigo et al., 2011; **Figure 1**). Thus, vernalization responsiveness appears to have evolved multiple times in response to selection by cold seasonal climates, and is hypothesized to have allowed expansion of clades within temperate zones (Preston and Kellogg, 2008; Kim et al., 2009; Edwards and Smith, 2010). For example, at least 3 out of 17 rosid, 5 out of 16 asterid, 1 out of 9 early-diverging eudicot, and 4 out of 10 monocot orders contain species that respond to vernalization. This conservative estimate suggests that vernalization responsiveness is a relatively evolvable trait at higher taxonomic levels. Whether this can be explained by relatively simple changes to pre-existing pathways is discussed below.

### GENETIC MODELS OF VERNALIZATION RESPONSIVENESS

In *A. thaliana*, vernalization responsiveness is mediated through epigenetic silencing of the flowering repressor gene *FLC*, and possibly its five *MADS AFFECTING FLOWERING* (*MAF*) paralogs, by the Plant-HomeoDomain-Polycomb Repressive Complex 2- (*PHD-PRC2*) complex (Ratcliffe et al., 2003; Kim et al., 2009). The *PHD-PRC2* complex initiates trimethylation of histone 2 lysine 27 (H3K27me3) and becomes progressively localized to the first intron of *FLC* during exposure to cold (Shindo et al., 2006; Angel et al., 2011; Strange et al., 2011). Recent evidence suggests that the mechanism for *PHD-PRC2* recruitment to *FLC* is associated with the *FLC* locus itself (Heo and Sung, 2010). Two non-coding transcripts that initiate from the first intron [COOL ASSISTED INTRONIC NONCODING RNA (COLDAIR)] and 3′-UTR (COOLAIR) of *FLC* are up-regulated in response to cold, and negatively regulate the transcription of *FLC* through recruitment of PRC2 (Heo and Sung, 2010; reviewed in Ietswaart et al., 2012). Reduction of *FLC* transcription in response to vernalization results in the release of *FT* and *SOC1* from negative regulation, permitting the shoot apex to respond to inductive flowering signals (Searle et al., 2006). Following cold treatment, warm temperatures and long days promote the expression of *FT* through the photoperiod, temperature-, and age-dependent pathways. This results in an *FT*-mediated morphological shift in the shoot apex from vegetative to inflorescence identity, via induction of MADS-box genes such as *FRUITFULL* (*FUL*) and *APETALA1* (*AP1*), and the eventual production of flowers, fruits (siliques), and seeds (reviewed in Adrian et al., 2009; Amasino, 2010).

Several members of the temperate grass subfamily Pooideae also respond to vernalization. However, since the ancestor of grasses was likely tropical, vernalization responsiveness in pooids is inferred to have evolved independently from vernalization responsiveness in the Brassicaceae (Clayton and Renvoize, 1986; Davis and Soreng, 2008; Preston and Kellogg, 2008; Edwards and Smith, 2010). In the closely related crop species wheat and barley (Pooideae), differences in vernalization responsiveness are largely a result of variation at three major loci: *VERNALIZATION1* (*VRN1*), *VRN2*, and *VRN3* (reviewed in Trevaskis et al., 2007a; Distelfeld et al., 2009). However several other genes are implicated in the pathway (Greenup et al., 2011). *VRN1* is homologous to the flower development genes *AP1*, *CAULIFLOWER *(*CAL*), and *FUL* in *A. thaliana*, and its expression is progressively induced during long durations of cold in response to vernalization-induced changes to chromatin at the *VRN1* locus (Oliver et al., 2009; Alonso-Peral et al., 2011). In wheat and barley cultivars that respond to vernalization, *VRN1* expression is repressed prior to winter by chromatin modifications mediated by proteins that interact with regulatory sites within the promoter or first intron. Simultaneously, the long day induction of *VRN1* is repressed by the zinc-finger *CO*-like gene *VRN2* (Hemming et al., 2009). Independent of *VRN1* expression, another MADS-box genes, *ODDSOC2*, is repressed during exposure to cold, resulting in the loss of transcriptional inhibition of downstream flowering genes (Greenup et al., 2010). Expression of *VRN1* is required for long-term repression of *ODDSOC2* and *VRN2* (Trevaskis et al., 2006; Hemming et al., 2008), ODDSOC2 negatively regulates the flower development gene *FLOWERING PROMOTING FACTOR 1* (*FPF1*; Greenup et al., 2010), and *VRN2* negatively regulates the temperate cereal *FT* ortholog *VRN3* above a certain threshold (Yan et al., 2004, 2006).

### EVOLUTION OF VERNALIZATION RESPONSIVENESS

**FIGURE 4:**
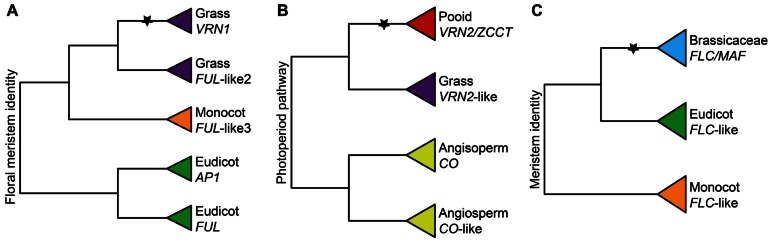
**Importance of gene duplications for the functional evolution of vernalization responsive genes.** **(A)** Simplified phylogeny of the *APETALA1/FRUITFULL* (*AP1/FUL*) gene family showing the grass-specific duplication that gave rise to *VRN1* based on Litt and Irish (2003) and Preston and Kellogg (2006). **(B)** Simplified phylogeny of the CCT zinc-finger gene family showing the pooid-specific duplication that gave rise to *ZCCT1* and *ZCCT2* based on Yan et al. (2004). **(C)** Simplified phylogeny of the *FLOWERING LOCUS C* (*FLC*) gene family showing the Brassicaceae-specific duplication that gave rise to *FLC* and *MAF *genes based on Becker and Theissen (2003). Inferred ancestral functions are stated at the base of each tree. Stars denote important duplication events for the evolution of VR. Pooid clades are red, grass clades are purple, monocot clades are orange, Brassicaceae clades are blue, eudicot clades are green, and angiosperm clades are yellow.

Vernalization responsiveness has evolved independently in several plant lineages, presumably in response to climate cooling events over the past 47.5 million years. Comparative genetic studies in a range of different angiosperm species suggest that vernalization responsiveness has evolved primarily through the neo-functionalization of ancient photoperiod pathway genes, including *CO*-like (i.e., *VRN2*), *FT*-like, *FUL*-like (i.e., grass *VRN1*), and *SOC1*-like (i.e., *FLC*), following their duplication (**Figure 4**). For example, phylogenetic analyses suggest that the *FLC*-like gene clade of *A. thaliana* is restricted to the Brassicaceae (Becker and Theissen, 2003; Ballerini and Kramer, 2011; **Figure 4**). Furthermore, although *FLC/MAF*-like genes are found in other core eudicots, gene expression tends to be positively rather than negatively regulated by cold. This is the case for *Arabidopsis MAF5*, the Texas bluebell (*Eustoma grandiflorum*, Gentianaceae) *EgFLCL*, and trifoliate orange (*Poncirus trifoliate*, Rutaceae) *PtFLC* (Zhang et al., 2009; Nakano et al., 2011). In *A. thaliana*, natural variation in vernalization responsiveness has been linked to variation in the promoter, first exon, and first intron of *FLC*, and within the positive regulator of *FLC*, *FRIGIDA* (*FRI*; Michaels and Amasino, 1999, 2001; El-Din El-Assal et al., 2001; Werner et al., 2005; Balasubramanian et al., 2006; Angel et al., 2011; Bond et al., 2011; Coustham et al., 2012; Wollenberg and Amasino, 2012). A similar role has been afforded to the *FLC* ortholog *PERPETUAL FLOWERING 1* (*PEP1*) in the Brassicaceae species *Arabis alpina*. However, since *Arabis alpina* is a perennial species, cold-induced chromatin modification of *PEP1* is only transient, being reset every growing season (Wang et al., 2009a).

In sugar beet (*B. vulgaris*, Amaranthaceae, Caryophyllales) recruitment of a lineage-specific *FT*-like gene duplication has been implicated in the independent origin of vernalization responsiveness (Pin et al., 2010; Mutasa-Gottgens et al., 2012). Under warm conditions, the *FT* homolog *BvFT1* represses its paralog *BvFT2*, resulting in a block to flowering. By contrast, under cold conditions, *BvFT1* is down-regulated, causing derepression of *BvFT2* and promoting flowering (Pin et al., 2010). It was recently discovered that down-regulation of *BvFT1* during winter is due to the repressive action of *BOLTING TIME CONTROL 1* (*BvBTC1*), which is related to the circadian clock *PRR* genes in *A. thaliana* (Pin et al., 2012). Moreover, variation in the response of *BvBTC1* alleles to vernalization has been linked to growth habit differences in domesticated sugar beet (Pin et al., 2012). Together with the fact that *VRN1* and *VRN2* are inferred to have evolved somewhere at the base of (Preston and Kellogg, 2006) or within (Yan et al., 2004) the Poaceae, respectively, these data suggest that lineage-specific flowering gene duplications have been important for independent origins of vernalization responsiveness, either through subtle switches from flowering inducers to repressors (e.g., *VRN2*, *FLC*, and *BvFT1*) or more dramatic changes in regulation and downstream targeting (e.g., *VRN1* and *BvBTC1*; **Figure 3**).

### GENETIC LINKS BETWEEN SEASONAL ADAPTATIONS TO COLD

Given the temporal overlap between endodormancy and cold acclimation, an interesting question is whether these two processes are linked at the genetic level. Presently, such links can be tentatively formed by combining data from cold acclimating but not endodormant *A. thaliana*, and endodormant trees. In the case of *A. thaliana*, CO is a regulator of both *FT* and *SOC1*. Similar to *FT*, *SOC1* positively regulates the expression of meristem identity genes, such as *FUL* and *LFY*. However, *SOC1* also negatively regulates the cold responsive *CBF* genes (Seo et al., 2009; **Figure 3**). Thus, although it needs to be explicitly tested in endodormant species, these data suggest a mechanism by which the break of endodormancy can influence the loss of cold acclimation.

Similar genetic associations can also be postulated for cold acclimation and vernalization responsiveness. Recent studies have shown that wheat *VRN1* has CBF-binding sites in its promoter, and that *VRN1* negatively regulates CBF genes (Alonso-Peral et al., 2011). This suggests a negative feedback loop between cold acclimation and vernalization. However, it remains to be tested whether CBF proteins actually bind to the *VRN1* promoter, and if so, whether this interaction is positive or negative. Interestingly, in *A. thaliana* genetic evidence suggests that CBF proteins positively regulate *FLC* in the autumn, accentuating the repression of flowering over winter (Seo et al., 2009; **Figures 2** and **3**). If this connection also exists in pooid grasses we would predict that CBF proteins positively regulate repressors of flowering such as *VRN2* and homologs of *SVP*. Consistent with this, it has been demonstrated that the barley *SVP*-like genes *Barley MADS1* (*BM1*), *BM10*, and *Vegetative to Reproductive Transition gene 2* (*VRT2*), and wheat *VRT2* are up-regulated by cold (Trevaskis et al., 2007b; Sutton et al., 2009; but see Kane et al., 2005). Alternatively, CBF proteins could directly repress flowering by negatively regulating *VRN1* or *VRN3*.

## CONCLUSIONS AND FUTURE PROSPECTS

Low to freezing temperatures are major determinants of latitudinal and altitudinal ranges of plants (Cavendar-Bares et al., 2005), and less than half of angiosperm plant families are distributed in regions with seasonally low temperatures (Ricklefs and Renner, 1994; Larcher, 2005). In the next 80 years it is predicted that global temperatures will increase by 1.1–6.4°C, and that there will be an increase in the frequency and/or severity of warm spells during winter months (USGCRP, 2009; National Research Council, 2010). Both these escalating intermittent temperature fluctuations and long-term climate changes have the potential to affect the phenology of cold temperate adapted species that rely on an extended period of cold for timely flowering (Loik et al., 2004; Offord, 2011). However, flowering time will ultimately result from the interaction of different genetic pathways in response to environmental factors such as photoperiod, cold, heat, water-stress, and developmental age (Cook et al., 2012). These interactions are only starting to be worked out.

Contrary to the hypothesis that cold-induced traits are hard to evolve, phylogenetic analyses in combination with past climate and trait data suggest multiple independent origins of cold acclimation, endodormancy, and vernalization responsiveness, at least in angiosperms. Furthermore, although often correlated, cold acclimation and endodormancy/vernalization responsiveness can be uncoupled at the physiological level, potentially allowing increased flexibility in species-specific responses to seasonal cold. Does this imply parallel evolution of the same ancestral genes and/or pre-adapted genetic pathways?

Available data support the hypothesis that cold-induced traits have evolved multiple times independently through the modification of the same genetic pathways. This suggests that these pathways are somewhat pre-adapted to providing avoidance of or tolerance to cold stress. However, the exact genes and proteins that have been recruited to cold adaptive traits differ from clade to clade. Many of the known key regulators of cold-induced physiological traits are members of large gene families that have broadly conserved roles in stress responses (e.g., dehydrin proteins) and/or developmental transitions (e.g., *VRN1/FUL*-like genes). In Pooideae, Brassicaceae, and poplar several genes involved in cold acclimation (e.g., *CBF/DRE* genes and *LRR*-containing genes), vernalization responsiveness (*CO*-like and *FLC*), and endodormancy (*FT*-like genes) have evolved from lineage-specific duplications (Becker and Theissen, 2003; Yan et al., 2004; Sandve et al., 2008; Sandve and Fjellheim, 2010; **Figure 4**). Loss of vernalization responsiveness has been documented for multiple cultivars of wheat and barley under artificial selection either through the loss of *VRN2*, or the loss of negative-*cis*-regulatory elements in *VRN1* and *VRN3* (Yan et al., 2003, 2004; Fu et al., 2005; Andersen et al., 2006; Szucs et al., 2007; Schwartz et al., 2010; Alonso-Peral et al., 2011). Whether these changes can happen rapidly enough in natural populations to combat human-induced climate change is a hot topic of debate.

Despite an unfolding picture at the broad phylogenetic scale that suggests multiple evolutionary origins of cold adaptive traits (**Figure 1**), relatively little physiological, developmental, or genomic data are available for understanding the evolutionary lability of seasonal cold adaptations at the family level and below. This is particularly true for species that might have retained cold adaptive traits following secondary shifts to the tropics, thus hampering our understanding of adaptation on relatively short timescales. Nonetheless, exciting recent and ongoing experimental and phylogenomic studies in both tropical and temperate taxa of the Brassicaceae (Brassicales), Poaceae (Poales), Pinaceae (Pinales), and Phrymaceae (Lamiales) are providing novel insights into the tempo, ancestral selection pressures, and potential constraints related to the evolution of cold acclimation/tolerance, endodormancy, and vernalization responsiveness (e.g., Lin et al., 2005; Preston and Kellogg, 2008; Sandve and Fjellheim, 2010; Méndez-Vigo et al., 2011; Sandve et al., 2011; Friedman and Willis, 2013; Humphreys and Linder, 2013). Successful studies will need to combine physiological observations with ancestral state reconstruction and genetic/genomic analyses to determine the direction of trait shifts, and any prerequisites for their evolution. Finally, population-level studies will continue to provide insight into evolutionary responses to more subtle (both temporal and quantitative) seasonal variation in temperature across species’ ranges.

## Conflict of Interest Statement

The authors declare that the research was conducted in the absence of any commercial or financial relationships that could be construed as a potential conflict of interest.
